# Growth patterns in children and adolescents with cerebral palsy from Argentina and Germany

**DOI:** 10.1038/s41598-023-34634-6

**Published:** 2023-06-02

**Authors:** Maria de las Mercedes Ruiz Brunner, Eduardo Cuestas, Rüdiger von Kries, Jordan Brooks, Charlotte Wright, Florian Heinen, Andreas Sebastian Schroeder

**Affiliations:** 1 Instituto de Investigación en Ciencias de la Salud, Universidad Nacional de Córdoba, Consejo Nacional de Investigaciones Científicas y Técnicas, Córdoba, Argentina; 2 Department of Paediatric Neurology and Developmental Medicine, Hauner Children’s Hospital, Ludwig Maximilian University of Munich (LMU), Munich, Germany; 3 Catedra de Clínica Pediátrica, Facultad de Ciencias Médicas, Hospital Nuestra Señora de La Misericordia, Universidad Nacional de Córdoba, Córdoba, Argentina; 4 Division of Epidemiology, Institute of Social Pediatrics and Adolescent Medicine, LMU University, Munich, Germany; 5 Life Expectancy Project, San Francisco, CA USA; 6 Royal Hospital for Children, Glasgow Royal Infirmary, Glasgow, UK

**Keywords:** Nutrition, Paediatric research

## Abstract

To analyze growth patterns of children with CP between countries; to examine differences in growth; and to assess the fit of growth charts. Cross-sectional study in children with CP from 2 to 19 years old, 399 from Argentina and 400 from Germany. Growth measures were converted into z-scores and compared to WHO reference and US CP growth charts. Generalized Linear Model was used to analyze the growth expressed as mean z-scores. 799 children. Mean age 9 years (± 4). Compared to the WHO reference, the decrease in Height z-scores (HAZ) with age in Argentina (− 0.144/year) was double that in Germany (− 0.073/year). For children in GMFCS IV–V, BMI z-scores (BMIZ) decreased with age (− 0.102/year). Using the US CP charts, both countries showed decreasing HAZ with age, in Argentina (− 0.066/year) and in Germany (− 0.032/year). BMIZ increased more among children with feeding tubes (0.062/year), similar in both countries. Argentinian children with oral feeding decrease their Weight z-score (WAZ) by − 0.553 compared to their peers. With WHO charts BMIZ presented an excellent fit for GMFCS I–III. HAZ presents a poor fit to growth references. BMIZ and WAZ presented a good fit to US CP Charts. Growth differences due to ethnicity also act in children with CP, and are related to motor impairment, age and feeding modality, possibly reflecting differences in environment or health care.

## Introduction

Anthropometric assessments consist of taking anthropometric measurements and comparing them with growth standards to assess growth. In a population, this comparison could be used to measure overall well-being at a given point in time, taking into account the age and sex of the children^1^.

Growth in typically developing (TD) children varies between countries. When comparing healthy economically-privileged children from non-European countries with those from northern European countries, the latter tend to be 5 cm taller than the WHO reference population, while non-European children fall to approximately 5 cm below the reference^2,3^. Since the children included in the mentioned study were all considered to be relatively economically privileged, it has been suggested that these differences in growth may relate to inherent differences^2^. However other evidence suggests that these differences are likely to be environmental in origin^4,5^, as population height differences have been observed to diminish over time as populations become even more affluent^6^. Differences in normal growth across countries may also need to be considered when assessing growth in children with chronic diseases that affect development.

Children with cerebral palsy (CP) have delayed growth and tend to be shorter and lighter than TD peers. The divergence from normal growth patterns is greatest for children with severe motor impairment and increases with age^7,8^. Additionally, feeding difficulties, malnutrition, low growth hormone levels, and lower levels of physical activity have also been related to growth restriction^9–11^.

Growth charts derived from more than 100,000 growth measures in 25,545 US children with cerebral palsy have been published^12^. Subsequent studies from other countries have reported that these charts provide a reasonable basis for monitoring growth of children with CP from Brazil and the UK^13,14^. In particular, growth assessments of British children fitted well with the US charts regarding weight-for-age and body mass index (BMI)-for-age. British children with CP, however, were taller than the US CP growth reference^14^. Whether this difference varied with age or other characteristics (such as sex or feeding modality) was not reported in detail.

To our knowledge, differences in growth between European and South American children and adolescents with cerebral palsy have not been previously studied. The aims of the present study are: (1) to analyze and compare the growth patterns of children with CP between countries to see if the population growth patterns of each country are repeated despite CP condition; (2) to examine differences in growth with respect to age, severity of motor impairment and feeding modality and (3) To assess the fit of WHO and US CP growth reference charts in Argentinian and German children with CP.

## Method

This was a cross sectional study, with data collected retrospectively. Children with confirmed diagnosis of CP were included when weight and height measurements and diagnosis information were available. The case definition of CP developed by the Surveillance of Cerebral Palsy in Europe (SCPE) and international references was used in both countries^15,16^. Those with a stated genetic or metabolic syndrome, (e.g. Angelman syndrome, Chromosomal aberration, etc.) potentially affecting growth (n = 40), or had incomplete information (n = 71) were excluded (Supplementary Fig. 1).

The sample was hospital-based, including as much cases as were possible. Information on the Argentinian CP sample was obtained from the database of the INICSA (Instituto de Investigaciones en Ciencias de la Salud). Anthropometric measurements were taken from children from five Argentinian cities (Cordoba, Ciudad Autonoma de Buenos Aires, Catamarca, Santiago del Estero and Jujuy) as part of a multicenter research study during years 2016 to 2018. Consecutive sampling was implemented in each institution, including as many children with CP as possible. Children were excluded when they did not fulfill the inclusion criteria requirements or were not available at the moment of data collection.

The German CP sample was obtained from medical records at the iSPZ Hauner (University Children’s Hospital, Munich). Data were collected from children that had received medical care between 2012 and 2019. For each child only the last complete anthropometric assessment was used for comparison. All children with complete anthropometric information were included and consecutive sampling was performed. Demographic characteristics, distribution of phenotype of CP (unilateral vs. bilateral pattern), severity of motor impairment (GMFCS levels I–V), oral feeding versus non-oral (non-oral included children fed totally or partially with a feeding tube), and anthropometric measurements (weight and height) were obtained from the medical records of each child.

The present study was conducted in accordance with the Good Clinical Practice and the Declarations of Helsinki. It was approved by the local ethics committee of the University Children’s Hospital, Ludwig-Maximilians-Universität (LMU) (No. 18-759) in Germany, and by the Ethical Evaluation Board of Health Research (COEIS) of the province of Cordoba (REPIS No. 3262/3236) in Argentina.

### Anthropometric assessment

International standards for anthropometric assessment were followed^17^. Anthropometric measurements were collected from trained health professionals. Weight and height were collected using direct methods while children were wearing light clothes and no shoes. All measurements were taken twice and the average measurement was used for analysis.

Weight was obtained in kilograms to the nearest 100 g using a wheelchair scale or a digital scale, depending on the child’s abilities. In this study, the term height was used to refer to both height and length. Height was measured depending on the ability of the child to stand. When the child could not stand, length was measured in the supine position. Height was taken twice; if there was a difference of more than 1 cm between the two measurements, the data were excluded and the previous record of completed measure was included (n = 23). When direct height could not be obtained, it was not included in the medical records. If knee height was available, height was estimated with published equations for children with cerebral palsy using knee height when this segmental measure was available (n = 31, 3.9%)^18^.

Anthropometric measures were converted into z-scores to be compared considering sex and age, and were used to describe how far a measurement is from the median (average) of a reference population. z-scores for Argentinian and German children with CP were calculated based on international growth references for TD children from WHO (2007) for BMI and height^19^. Weight-for-age was not included from references for TD children as there is no information of weight-for-age for children older than 10 years old from the WHO charts. The WHO Multicenter Growth Reference Study can be used worldwide as there were designed to provide data describing standard for normal growth (how children *should* grow), by including in the study’s selection criteria with recommended health behaviors^19^.

To consider local population references, anthropometric measurements were converted into z-scores according to reference populations. z-scores for Argentinian children with CP were calculated based on local growth references for TD children from Argentina (Sociedad Argentina de Pediatría (SAP)^20^). z-scores for German children with CP were calculated using local reference charts from Germany (Gesundheit von Kindern und Jugendlichen in Deutschland (*KiGGS*)^21^).

Also, z-scores for both the Argentinian and German children with CP were calculated based on the US CP growth charts for BMI, height- and weight-for-age using the procedure outlined in the UK study of Wright, Cole et al. together with LMS growth software^12,14^. The fit of the growth curves was analyzed considering categories established in previous publications^14,22^, where mean z-score values ≤ 0.17 SD were classified as an excellent fit, within > 0.17 to ≤ 0.33 SD a good fit, and > 0.67 SD a poor fit. Weight-for-age z-scores were not used as they were not available for children older than 10 years of age from WHO Growth Charts (2007).

### Statistical analysis

The normality of the continuous data was tested using the Kolmogorov–Smirnov test. Summary statistics were presented as mean with SD or medians with interquartile range (IQR) and absolute or relative frequency (percentage) with 95% confidence intervals.

Anthropometric measurements of weight, height and BMI were converted into z-score values adjusted to age and sex of each individual, considering values from growth charts. z-score are used to describe how far a measurement is from the median, and can be used as a continuous number. z-score were calculated with the follow Equation^23^:

$${\text{z}} - {\text{score}} = \frac{{\left( {{\text{observed }}\,{\text{value}}} \right) - \left( {{\text{median}}\,{\text{ reference}}\,{\text{ value}}} \right)}}{{{\text{z}} - {\text{score}}\,{\text{ of}}\,{\text{ the }}\,{\text{reference }}\,{\text{population}}}}$$

A bivariate analysis was performed between the z-scores for anthropometric measurements of HAZ and BAZ for WHO growth charts and WAZ, HAZ and BAZ for US CP charts, compared to variables such as sex (female vs. male), GMFCS (level I to III vs. Level IV and V), country (Argentina vs. Germany), method of nutritional intake (oral vs. non-oral), and age group (divided into two age groups: 2–10 years and 11–19 years), using t-tests. Variables with *p* < 0.05 in bivariate analysis were considered for inclusion in the multivariate analysis.

Generalized Linear Models were used to analyze the association between growth expressed as mean z-score and all relevant clinical covariates. Main effects models were used to evaluate z-score change with full adjustment (controlling for all significant covariates). We fitted interaction models to evaluate effect modification between variables found to be statistically significant in main effects models. The final models were chosen to have the best fit statistics defined by the lowest restricted maximum likelihood. Statistical significance was set at *p* < 0.01.

All analyses were performed by using IBM SPSS statistical software, V. 25 (IBM Corp, Armonk, New York, U.S.A.). Multivariable comparison graphs were developed using MedCalc V19.0 statistical software.

### Ethical approval

The study was conducted in accordance with the Declarations of Helsinki. Approval for the study was given by Hauner’s Children Hospital ethical committee, Ludwig-Maximilians-Universität (LMU) Institutional Review Board (No. 18-759) and the Ethical Evaluation Board of Health Research (COEIS) of the province of Cordoba (REPIS No. 3262/3236). The privacy, confidentiality and security of the participants’ personal data were safeguarded. The research was registered in the DRKS – German Clinical Trial Register (DRKS00016407, Date of Registration in DRKS: 2018/12/21).

### Consent to participate

The need for patients’ written consent was deemed unnecessary by the institutional review boards as we did not contact the families to conduct this retrospective study.

## Results

There were 799 growth measurements, 399 from Argentinian children with CP and 400 German children with CP. The mean age was 9 years and 5 months (SD 4 years 7 months). The characteristics of the sample are presented in Table 1.z-scores of anthropometric measurements were assessed with national growth charts, WHO growth charts and US CP Charts (Table 1). Children with CP have lower weight, height and BMI than their peers in their home countries, with negative z-scores. z-scores were also compared according to gross motor function and country in Supplementary Table 1. Children with CP have lower weight, height and BMI than their TD peers in all GMFCS levels. When comparing to WHO international growth charts, Argentinian children with CP presented lower z-score values than their German peers (Table 1), also when GMFCS level is considered (Supplementary Table 1).

**Table 1:** Characteristics from Argentinian and german sample.

	Argentina (n = 399)		Germany (n = 400)	
	n		n	
**Sex % [95% CI]**				
Female	157	39.4 [34.5, 44.3]	168	42.0 [37.1, 47.0]
Male	242	60.6 [55.7, 65.4]	232	58.0 [53.0, 62.9]
**Age groups % [95% CI]**				
2 to 5 years	86	21.5 [17.6, 25.9]	138	34.8 [29.8, 39.4]
6 to 10 years	127	31.8 [27.3, 36.6]	152	38.0 [33.2, 42.9]
11 to 14 years	111	27.9 [23.5, 32.5]	65	16.2 [12.8, 20.2]
15 to 19 years	75	18.8 [15.1, 22.9]	44	11.0 [8.1, 14.5]
**Level of GMFCS % [95% CI]**				
Level I	63	15.9 [12.3, 19.7]	182	45.5 [40.5, 50.5]
Level II	55	13.8 [10.5, 17.5]	56	14.0 [10.7, 17.8]
Level III	60	15.0 [11.7, 18.9]	57	14.2 [11.0, 18.1]
Level IV	75	18.8 [15.1, 22.9]	74	18.5 [14.8, 22.6]
Level V	146	35.6 [31.8, 41.5]	31	7.8 [5.3, 10.8]
**Topography % [95% CI]**				
Unilateral CP	87	21.8 [17.8, 26.2]	120	30.0 [25.5, 34.7]
Bilateral CP	312	78.2 [73.8, 81.1]	280	70.0 [65.2, 74.4]
**CP type % [95% CI]**				
Spastic	306	76.7 [72.2, 80.7]	320	82.3 [75.7, 83.8]
Dyskinetic	29	7.3 [4.9, 10.3]	47	12.1 [8.8, 15.3]
Ataxic	17	4.3 [2.5, 6.7]	11	2.8 [1.4, 4.9]
Mixed	35	8.8 [6.2, 12.0]	11	2.8 [1.4, 4.9]
Not informed	12	3.0 [1.6, 5.2]	–	–
**Feeding % [95% CI]**				
Oral	356	89.2 [85.7, 92.1]	379	94.7 [92.1, 96.7]
Non-oral	43	10.8 [7.9, 14.2]	21	5.3 [9.3, 7.9]
**Anthropometric measures**				
Weight, kg				
Median [IQR]	399	23.9 [16.5,38.7]	400	22.7 [16.2,34.9]
Height, cm				
Mean [SD]	399	125.8 [23.4]	400	124.9 [23.9]
BMI				
Median [IQR]	399	15.9 [14.0,18.6]	400	15.6 [14.2,18.1]
**National growth charts**				
Weight for age z-score Mean [SD]	399	− 1.44 [2.32]	400	− 1.23 [1.82]
Height for age z-score				
Mean [SD]	399	− 1.16 [2.05]	400	− 1.11 [1.62]
BMI for age z-score				
Mean [SD]	^c^	^c^	400	− 0.69 [1.68]
**WHO growth charts**				
Weight for age z-score Mean [SD]	191^d^	− 1.26 [1.92]^d^	271^d^	− 0.52 [1.56]^d^
Height for age z-score Mean [SD]	399	− 1.96 [1.92]	400	− 0.59 [1.48]
BMI for age z-score Mean [SD]	399	− 0.79 [2.01]	400	0.37 [1.64]
**US CP chart**				
Weight for age z-score Mean [SD]	399	0.24 [1.06]	400	0.47 [0.89]
Height for age z-score Mean [SD]	399	0.52 [0.89]	400	0.95 [0.73]
BMI for age z-score Mean [SD]	399	− 0.29 [0.93]	400	− 0.37 [0.82]

*GMFCS* gross motor function classification system.

^a^Growth charts from the Argentine society of pediatrics^24^.

^b^Growth charts form the Robert Koch Institute (KiGGS)^21^.

^c^There are no national BMI charts available in Argentina.

^d^There is no information of weight-for-age for children older than 10 years old from the WHO charts.

To compare with their TD peers in the following analyses, we continue to use the WHO international growth charts that present an international average of growth in weight, height and BMI.

We performed a bivariate analysis between BMIZ and HAZ of the WHO Charts, compared to country, sex, GMFCS level, Feeding and Age, and between BMIZ, HAZ and WAZ of the US CP Chart compared to the same variables (Table 2). With WHO growth charts, HAZ presented differences in feeding, between countries, GMFCS levels and age. With US CP Charts, HAZ presented significant difference between countries, GMFCS level and age, but not regarding feeding (Table 2).

**Table 2:** Bivariate analysis of variables according to z-scores values (n = 799).

	WHO charts (2007)				US CP chart (2011)					
	BMIZ	*p* value	HAZ	*p* value	BMIZ	*p* value	HAZ	*p* value	WAZ	*p* value
Country	Mean (SD)		Mean (SD)		Mean (SD)		Mean (SD)			
Germany	0.37 [1.64]	**0.001**	− 0.59 [1.48]	** < 0.001**	− 0.37 [0.82]	0.197	0.95*** [0.73]	** < 0.001**	0.47 [0.89]	**0.001**
Argentina	− 0.79*** [2.01]		− 1.96*** [1.92]		− 0.29** [0.93]		0.52 [0.89]		0.24** [1.06]	
Sex										
Female	− 0.52 [1.73]	0.445	− 1.30*** [1.86]	0.740	− 0.28** [0.86]	0.168	0.76 *** [0.89]	0.448	0.40 [1.01]	0.237
Male	− 0.62 [1.92]		− 1.26*** [1.85]		− 0.37 [0.88]		0.72 *** [0.80]		0.32** [0.97]	
GMFCS										
Level I to III	− 0.13* [1.50]	** < 0.001**	− 0.60 [1.44]	** < 0.001**	− 0.31** [0.82]	0.363	0.82*** [0.78]	**0.001**	0.41 [0.92]	**0.056**
Level IV and V	− 1.21*** [2.10]		− 2.35*** [1.94]		− 0.37 [0.95]		0.61 [0.91]		0.27** [1.07]	
Feeding										
Oral	− 0.57 [1.83]	0.695	− 1.22 *** [1.83]	**0.010**	− 0.35 [0.87]	**0.031**	0.73*** [0.83]	0.401	0.32** [0.98]	**0.017**
Non-oral	− 0.67*** [2.06]		− 1.88 *** [1.92]		− 0.10*[0.89]		0.82 *** [0.90]		0.66 [1.05]	
Age										
2 to 10 years old	− 0.43 [1.78]	**0.004**	− 0.99 *** [1.76]	** < 0.001**	− 0.48 [0.84]	** < 0.001**	0.88 *** [0.83]	** < 0.001**	0.38[0.99]	0.341
11 to 19 years old	− 0.87***[1.93]		− 1.76 *** [1.89]		− 0.08* [0.89]		0.49 [0.81]		0.31** [0.98]	

Significant differences are marked in bold.

*BMIZ* BMI-for-age z-score, *HAZ* height-for-age z-score, *WAZ* weight-for-age z-score, *GMFCS* gross motor function classification system. Comparison to reference chart: *excellent fit (≤ 0.17 SD), **good fit (> 0.17 to ≤ 0.33 SD), ***poor fit (> 0.67 SD).

BMIZ presented significant differences between countries, GMFCS, and age when WHO growth references were used. Differences according to feeding and age were observed with US CP charts (Table 2).

With US CP Charts, WAZ presented significant difference between countries, GMFCS level and feeding, but not between age groups. Meanwhile, sex was not a significant variable for any z-score measurements and was not included in the multivariate analysis (Table 2).

### Analysis of fit to the two growth references

WHO growth charts overall presented a poor fit, with mean values for all sub categories well below the expected value, except for BMIZ younger children and those in GMFCS level I to III. With the US CP chart BMIZ presented an excellent to good fit for all sub categories, but mean values for HAZ were considerably higher than expected (Table 2).

### Multivariate comparison of BIMZ

The main effect model of the GLM for WHO’s reference charts showed that for BMIZ gross motor severity and age were variables that showed significant differences (*p* < 0.01), country and feeding did not show significant differences. The interaction effect model between GMFCS with age was significant for levels IV to V. In children with GMFCS level IV and V, BMIZ decreased − 0.102 z-score/per year of age (Table 3a).

**Table 3:** Generalized linear model for z-score in Argentinian and German children with cerebral palsy according to different growth charts (n = 799).

Model	Anthropometric measurement	Variables	β	95%CI	*p*
(a) WHO charts, 2007					
Main effect model	z-score BMI-for-age	Argentina, non-oral feeding, GMFCS IV and V	Reference		
		Germany	0.041	− 0.22 to 0.30	0.048
		Oral Feeding	− 0.440	− 0.90 to 0.02	0.063
		GMFCS I to III	1.105	0.84 to 1.37	** < 0.001**
		Age	− 0.037	− 0.06 to − 0.01	**0.007**
Interaction effect model	z-score BMI-for-age	GMFCS IV and V *age	− 0.102	− 0.13 to − 0.07	** < 0.001**
Main effect model	z-score Height-for-age	Argentina, non-oral feeding, GMFCS IV and V	Reference		
		Germany	0.852	0.62 to 1.08	** < 0.001**
		Oral Feeding	− 0.16	0.21 to − 0.57	0.451
		GMFCS I to III	1.338	1.10 to 1.58	** < 0.001**
		Age	− 0.061	− 0.08 to − 0.37	** < 0.001**
Interaction effect model	z-score Height-for-age	Germany*age	− 0.073	− 0.13 to − 0.10	**0.006**
		Argentina*age	− 0.144	− 0.18 to − 0.10	** < 0.001**
		GMFCS I to III*age	0.087	0.04 to 0.14	**0.001**
		Germany* GMFCS I to III	0.767	0.17 to 1.36	0.012
		Germany* GMFCS IV andV	0.233	− 0.34 to 0.81	0.428
		Argentina* GMFCS I to III	0.525	− 0.06 to 1.11	0.080
(b) US CP chart (2011)					
Main effect model	z-score BMI-for-age	Argentina, non-oral feeding, GMFCS IV and V	Reference		
		Germany	− 0.026	− 0.15 to 0.10	0.685
		Oral Feeding	− 0.306	− 0.53 to − 0.81	**0.008**
		GMFCS I to III	0.148	0.02 to 0.28	0.025
		Age	0.044	0.03 to 0.06	** < 0.001**
Interaction effect model	z-score BMI-for-age	Oral Feeding*age	0.042	0.03 to 0.05	** < 0.001**
		Non-Oral Feeding*age	0.062	0.04 to 0.08	** < 0.001**
Main effect model	z-score Height-for-age	Argentina, non-oral feeding, GMFCS IV and V	Reference		
		Germany	0.321	0.21 to 0.44	** < 0.001**
		Oral Feeding	− 0.222	− 0.43 to − 0.02	0.034
		GMFCS I to III	0.096	− 0.02 to 0.21	0.116
		Age	− 0.050	− 0.06 to − 0.04	** < 0.001**
Interaction effect model	z-score Height-for-age	Germany*age	− 0.032	− 0.05 to − 0.02	** < 0.001**
		Argentina*age	− 0.066	− 0.08 to − 0.05	** < 0.001**
Main effect model	z-score Weight-for-age	Argentina, non-oral feeding, GMFCS IV and V	Reference		
		Germany	0.198	0.05 to 0.34	**0.007**
		Oral Feeding	− 0.429	− 0.69 to − 0.17	**0.001**
		GMFCS I to III	0.120	− 0.03 to 0.00	0.115
		age	− 0.013	0.85 to 1.04	0.089
Interaction effect model	z-score Weight-for-age	Germany*Oral feeding	− 0.261	− 0.57 to 0.04	0.095
		Argentina* Oral Feeding	− 0.553	− 0.86 to − 0.24	** < 0.001**

Significant differences are marked in bold.

*GMFCS* gross motor function classification system.

**Figure 1:**
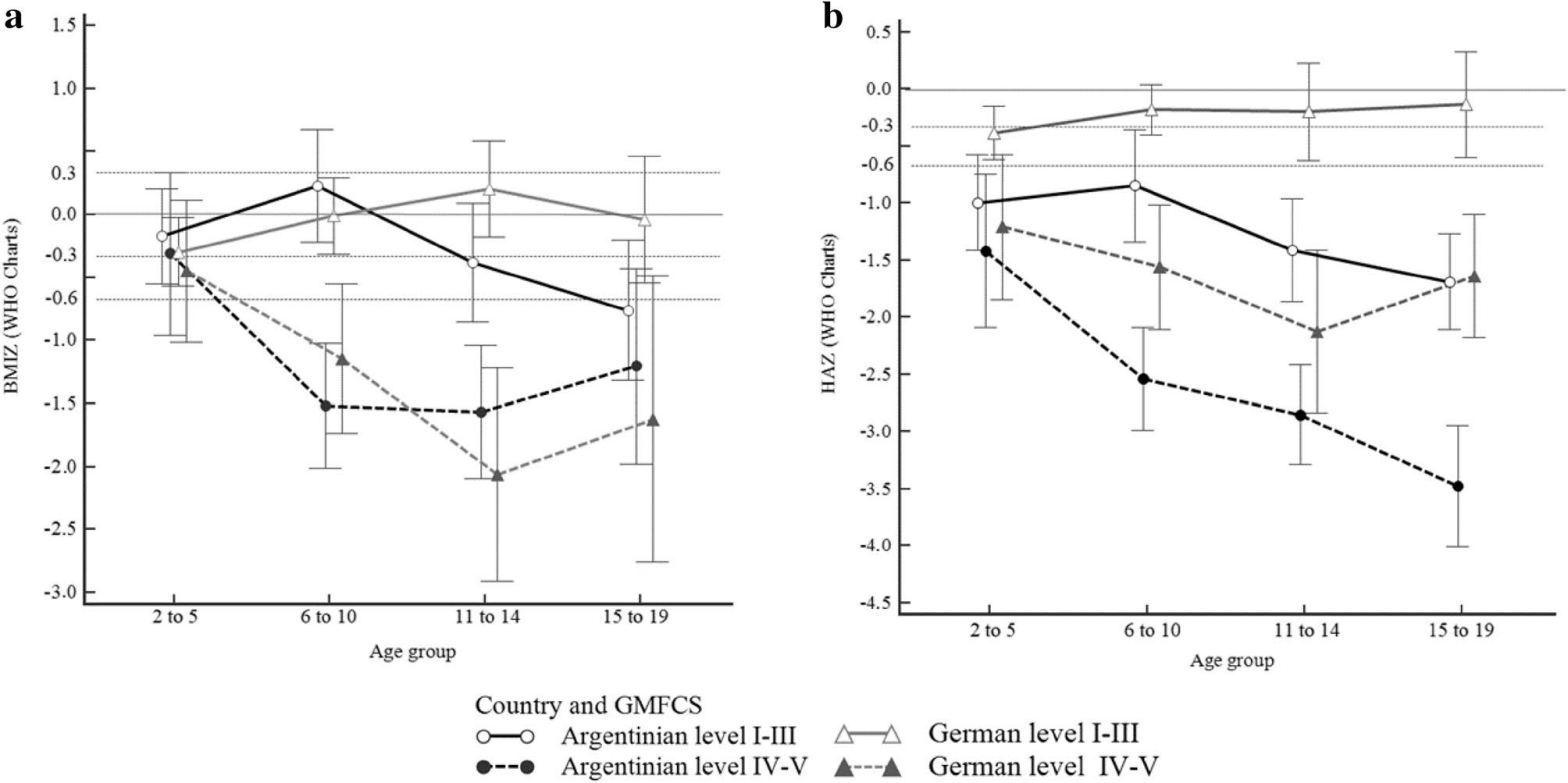
z-score (mean; 95% CI) comparison for anthropometric measures according to children with TD from WHO growth charts (2007) (n = 799). (**a**) BMI-for-age (BMIZ) by age considering country and GMFCS level for children with CP. (**b**) Height-for-age (HAZ) by age considering country and GMFCS level for children with. *GMFCS* gross motor function classification system. Dotted lines mark the thresholds for a good fit (< 0.33 SD) and a poor fit (> 0.67 SD) to the growth reference.

In Fig. 1a, it is observed that German and Argentinian children with CP and GRFCS level IV and V presented lower z-score values that their pears. Children with CP and GMFCS IV and V presented their lowest BMIZ at age 11 to 14 years old for both countries (Fig. 1a).

When BMIZ was analyzed considering US CP Charts. The main effect model of the GLM showed significant differences in feeding and age (*p* < 0.01). There were no differences in BMI growth between countries or GMFCS levels (Table 3b). The interaction effect model showed that as age increased children with oral feeding increased their BMIZ 0.042 z-score per year (*p* < 0.001), but children with non-oral feeding increased 0.062 z-score/per year (*p* < 0.001).

**Figure 2:**
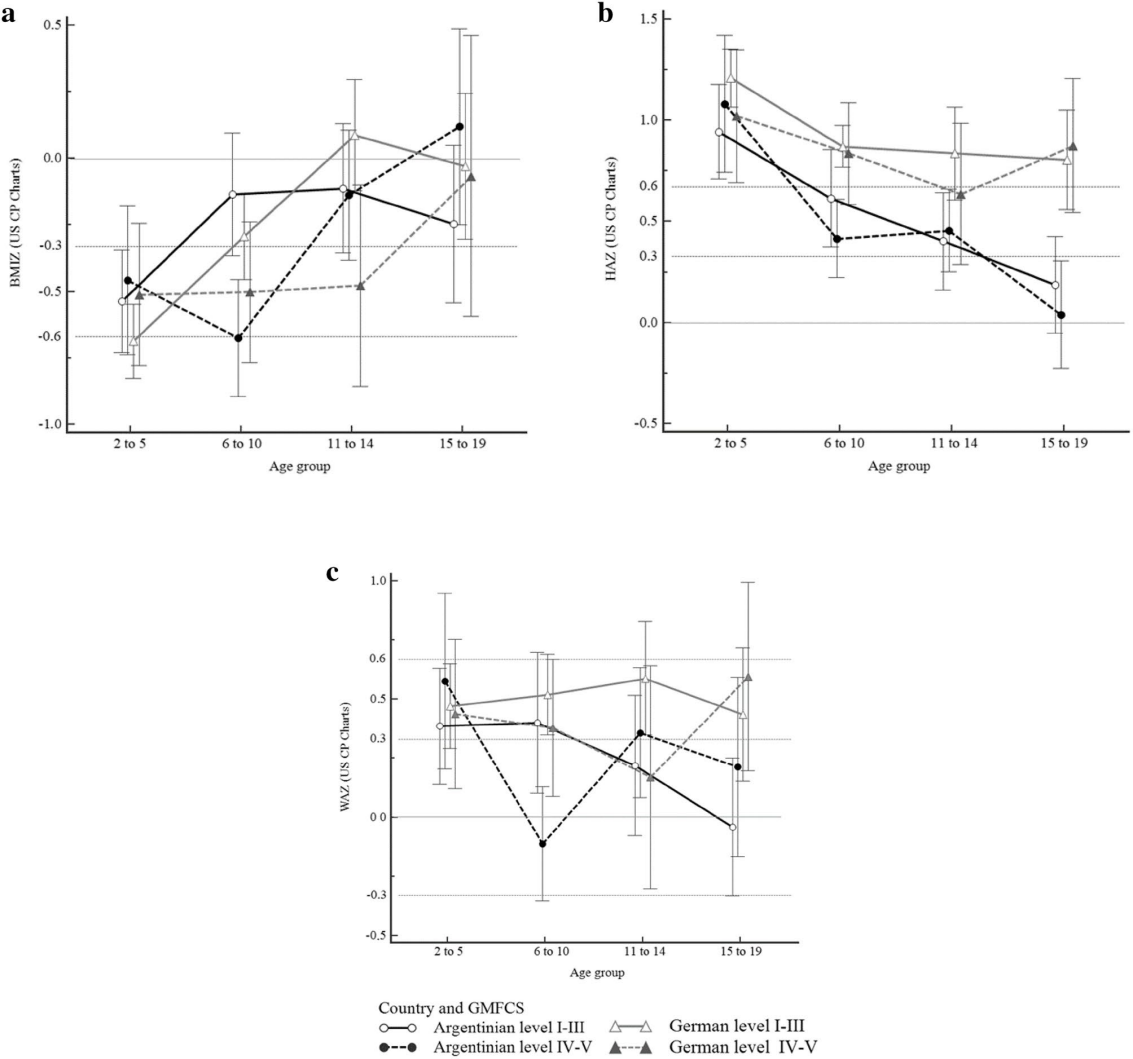
z-score (mean; 95% CI) comparison for anthropometric measures according to children with TD from US CP Charts (2011) (n = 799). (**a**) BMI-for-age (BMIZ) by age considering country and GMFCS level for children with CP. (**b**) Height-for-age (HAZ) by age considering country and GMFCS level for children with CP. (**c**) Weight-for-age (WAZ) by age considering country and GMFCS level for children with CP. *GMFCS* gross motor function classification system. Dotted lines mark the thresholds for a good fit (< 0.33 SD) and a poor fit (> 0.67 SD) to the growth reference.

Figure 2a, showed that considering US CP Charts, BMIZ was less than 0 for most age groups in both countries.

### Multivariate comparison of mean HAZ

HAZ according to WHO growth references was analyzed with the Generalized Linear Model (GLM) adjusting to all significate variables in the bivariate analysis. The GLM showed in the main effect model significant differences between countries, GMFCS levels and age (*p* < 0.01) (Table 3a). The interaction effect model showed that as age increased, German children with CP had a − 0.073 z-score/per year decrease in HAZ compared with children with TD WHO growth references (*p* = 0.006). Meanwhile Argentine children decreased − 0.144 z-score/per year compared with children with TD WHO growth references (*p* < 0.001).

The Interaction effect model form the GLM, also showed significance between age and GMFCS level I to III. As age increase, children from both countries with CP and GMFCS I to III increased their HAZ by 0.087 z-score/year (*p* = 0.001).

In Fig. 1b, it is observed that children with cerebral palsy in Argentina tend to have lower HAZ than German children with cerebral palsy. As age increases, the differences between countries also increase.

Considering the US CP charts, HAZ in the main effect model of the GLM showed significance between country and age (*p* < 0.001). The Interaction effect model showed that German children decreased − 0.032 z-score/per-year compared to US CP charts (*p* < 0.001), and Argentinian children decreased − 0.066 z-score/per-year (*p* < 0.001). Therefore, as age increased, the HAZ decreased in both countries. Argentinian children with CP have lower HAZ than German children when US CP charts is considered (Table 3b).

Figure 2b showed that HAZ presented higher values than the US CP reference population, irrespective of the GMFCS level or country. German children tend to have higher HAZ than Argentinian children and US CP reference chart.

### Multivariate comparison of WAZ

WAZ was analyzed considering US CP Charts. The main effect model of the GML for US CP charts showed that WAZ presented significant differences between countries and feeding (*p* < 0.01). When the variables were combined in the interaction effect model, only Argentinian children with oral feeding presented significant differences (*p* < 0.001). Argentinian children with oral feeding decrease by − 0.553 z-score units (*p* < 0.001) of WAZ, compared to their peers with non-oral feeding.

It is observed in Fig. 2c, that WAZ was between 0 and 0.67 for both countries, showing values with good fit to the references of US CP charts.

## Discussion

This study, compared growth in children and adolescents with CP from a northern European (Germany) and a southern American country (Argentina) to both the WHO reference for TD children^19^, and the US CP references from Brooks et al.^12^.

Children with CP in both countries are shorter, lighter and smaller than their typically developing peers and these differences increase as their GMFCS level and age increase. Previous studies have also demonstrated that children with CP grow differently from their TD peers and that GMFCS level strongly influences their growth^10,12,14,25^. Studies from German and Argentinian children with CP have also recently shown this difference^26–28^.

The second main finding is that between the two countries, children with CP differed significantly in height. German children with CP with GMFCS level I to III grow similarly to their TD peers of the same age and sex, but those with GMFCS level IV and V are significantly shorter. In contrast, all the Argentinian children with CP were shorter than their TD peer, no matter the GMFCS level and their HAZ tended to decrease with age, twice as fast as for German children. Growth differences after pubertal age have been shown for healthy children in different countries and ethnicities^2,29^. A large meta-analysis on fifty-three different healthy populations indicated that the mean height of preadolescent healthy children differs by 3 to 5 cm. At puberty, most non-European populations fall approximately 5 cm below the reference and northern European populations exceeding the reference by a similar amount^2^. This finding may be explained proximally by population differences in the initiation and progression of puberty in TD children. In Argentina, children with TD show a 2-year earlier sexual development, which leads to an earlier closure of the epiphyseal growth plate and explains a shorter final height in the general population when is compared to international references^30,31^. Differences in the onset of puberty, which may also relate to the severity motor impairment, could partially explain differences in height between TD children and children with CP found in our study^11,32^. The present study did not measure pubertal status or progression in children with CP, and further research would be needed to examine this possibility.

Our study demonstrates, for the first time, that the same ethnic differences that can be observed in children with TD can also been seen in children with CP. Differences in height between countries are multifactorial, but are likely to mostly relate to epigenetic, and environmental rather than genetic, factors^33^. Secular trends in height are commonly seen within countries, related to changing socio-economic status, nutrition, and health, and these can serve as public health indicators for interactions between growth and environment^4,5^. It has been demonstrated that social and psychological factors (such as socioeconomic status, parental education or emotional deprivation) are related to linear growth, and the effects of socio-economic crisis can increase low birthweight prevalence and can affect secular changes^34–36^. The effect of environmental factors may also explain differences in growth in children with CP from countries with difference socio-economic realities and healthcare systems, such as Germany and Argentina. Further research is needed to understand how environmental factors affect growth in children with CP.

It is puzzling that US CP charts tended to underestimate height, with HAZ higher than the US CP charts for children with CP in both countries, a similar finding to the earlier UK study^14^. For context, it should be noted that height measurements used to develop the US CP charts were taken from medical records and were not validated, and the authors recommend that height curves should therefore be viewed with caution^8,12^. Due to the difficulties in measure height in children with CP with severe motor compromise, it is possible that the differences in height could be related to the differences in measurement methods. Beyond the possible bias, differences were greater than 0 HAZ z-score for all GMFCS levels. It is also possible that this reflects the fact that the data used for the US charts was collected longer ago and that children with CP may grow better now than previously, due to improvements in neonatal nutrition. Future prospective multicenter studies with training measurement methods could help adjust this bias.

Besides the ethnic and sociodemographic differences, further influencing variables need to be considered for children with a chronic disease (such as CP). Muscle involvement due to brain injury would explain the differences in growth between those children with TD and those with CP. Their muscles tend to be smaller, stiffer and weaker than typical muscles, which would affect growth^37^. On the other hand, compared to the US CP growth chart, children with non-oral feeding had higher BMIZ and a better fit to the charts. On the other hand, for WAZ there was a better fit for children with oral feeding, who presented lower z-score values than their peers with non-oral feeding. In the multivariate analysis, Argentinian children that were fed orally presented a significantly lower z-score than those with non-oral feeding. These findings suggest that some of the growth deficit in children with CP and GMFCS level V could be related to nutritional deficit, and when they are fed enterally, totally or partially, nutrition is more secure. This difference in growth according to the feeding modality have already been mentioned in other CP studies, showing differences between countries, and required the development of different growth charts according to feeding in the CP US Charts^12,38^.

From the results obtained in this study, it seems important to highlight that in Argentina better efforts should be made to detect CP at earlier ages, improve access to more intensive and frequent rehabilitation treatments. It seems certain that Argentine children with CP would benefit from early access to specialized nutritional assessment appropriate to the level of severity as well as economic support to improve food access. Inequities in access to public rehabilitation centers among lower-income families should also be seriously addressed through financial assistance for transportation and relief from lost work hours for parents. All this would bring great relief to families with improved quality of life. In Germany, the integration and access of a few cases of the most isolated populations in rural areas and of urban migrants’ populations should be further improved.

Some limitations should be mentioned. Selection bias may exist when comparing different study populations. In Argentina, we were able to observe that the centers included had more children with GMFCS levels IV–V, whereas in the German study site patients showed a broader range of GMFCS Levels, and was predominately Level I. The prevalence of less compromised motor impairments seems to be increasing in European countries and Australia^39–41^, with a similar distribution as that in our German sample. It is possible that in Argentina there is a bias due to the type of centers included, where motor disabilities were more severe. In the absence of a complete local register, it is difficult to establish if our sample distribution is representative of the Argentinian CP population. Another limitation is the possible information bias from a retrospective study. We tried to control this bias by including only patients with complete anthropometric measurements and chose their last visit if multiple measures were available. However, because of the difficulty of measuring height in children with CP, it is possible that children with more severe motor compromise were lost because measurements were not available. Due to the limitations of height measurement and the lack of relation between BMI and body fat mass^42,43^, the interpretation of BMI as an indicator of body composition should be considered with caution. To study nutritional assessment in detail, other anthropometric measures beyond weight, height and BMI are recommended^44^ to assess nutritional status, such as segmental measures and skin fold. These measures were not available in all medical records.

The major strength of this study was the capacity to monitor growth in two well controlled settings. Another strength is the chance to compare children with CP form two difference settings, Argentina and Germany. Our analysis of growth possibly reflects environmental and health care differences that should be deeply studied in the field.

## Conclusion

Differences in growth pattern between Argentinian and German children with CP exist, and may be related to environmental factors. German children with CP tend to be taller than Argentinian children. All Argentinian and German children with GMFCS level IV and V tend to be smaller and shorter than their peers with TD from the WHO international references and as age increases, differences increase. When US CP charts are used, it should be considered that BMIZ presents a good fit, but that HAZ should be considered with caution.

## Supplementary Information

Supplementary Information 1.

Supplementary Information 2.

## Data Availability

Pseudonymized participant data reported in this article can be shared in compliance with current data protection regulations by the European Union. Data sharing requires a current and positive vote by the requestor’s competent ethics committee. All data will be available on reasonable request from corresponding author.

## Abbreviations

BMIZ: Body mass index for age z-score
HAZ: Height for age z-score
WAZ: Weight for age z-score
