# Supplementary material for: Growth patterns in children and adolescents with cerebral palsy from Argentina and Germany
Source: Sci Rep. 2023 Jun 2;13:8947. doi: 10.1038/s41598-023-34634-6 (PMC10238482; doi:10.1038/s41598-023-34634-6)
Supplement: Supplementary file 1 — Supplementary Information 1. [file 41598_2023_34634_MOESM1_ESM.docx]

Supplementary Figure 1 - Flow chart of patient’s inclusion in the study

German sample

Argentinian sample
