# Supplementary material for: Growth patterns in children and adolescents with cerebral palsy from Argentina and Germany
Source: Sci Rep. 2023 Jun 2;13:8947. doi: 10.1038/s41598-023-34634-6 (PMC10238482; doi:10.1038/s41598-023-34634-6)
Supplement: Supplementary file 2 — Supplementary Information 2. [file 41598_2023_34634_MOESM2_ESM.docx]

Supplementary Table 1. Z-score values for anthropometric measurements according to gross motor function classification system (GMFCS) and country.

|  | Argentina  (n = 399) | | Germany  (n = 400) | |
| --- | --- | --- | --- | --- |
|  | n | Mean [SD] | n | Mean [SD] |
| a. WHO growth chart |  |  |  |  |
| GMFCS Level I |  |  |  |  |
| *Weight for age z-score^a^* | 30 | -0.11 [1.57] | 122 | 0.03 [1.41] |
| *Height for age z-score* | 63 | -0.80 [1.61] | 182 | 0.08 [1.18] |
| *BMI for age z-score* | 63 | 0.04 [1.38] | 182 | 0.02 [1.45] |
| GMFCS Level II |  |  |  |  |
| *Weight for age z-score^a^* | 31 | -0.32 [1.25] | 38 | -0.31 [1.22] |
| *Height for age z-score* | 55 | -0.91 [1.24] | 56 | -0.49 [1.11] |
| *BMI for age z-score* | 55 | -0.18 [1.52] | 56 | -0.12 [1.46] |
| GMFCS Level III |  |  |  |  |
| *Weight for age z-score^a^* | 28 | -1.35 [1.52] | 41 | -0.86 [1.47] |
| *Height for age z-score* | 60 | -1.87 [1.51] | 57 | -1.07 [1.16] |
| *BMI for age z-score* | 60 | -0.55 [1.73] | 57 | -0.38 [1.50] |
| GMFCS Level IV |  |  |  |  |
| *Weight for age z-score^a^* | 29 | -1.01 [2.37] | 50 | -1.37 [1.59] |
| *Height for age z-score* | 75 | -2.26 [2.13] | 74 | -1.58 [1.47] |
| *BMI for age z-score* | 75 | -0.53 [2.11] | 74 | -1.18 [1.83] |
| GMFCS Level V |  |  |  |  |
| *Weight for age z-score^a^* | 73 | -2.21 [1.78] | 20 | -1.45 [1.71] |
| *Height for age z-score* | 146 | -2.73 [1.89] | 31 | -1.51 [2.02] |
| *BMI for age z-score* | 146 | -1.61 [2.18] | 31 | -1.11 [1.92] |
| a. National growth chart |  |  |  |  |
| GMFCS Level I |  |  |  |  |
| *Weight for age z-score* | 63 | -0.13 [1.61] | 182 | -0.44 [1.41] |
| *Height for age z-score* | 63 | 0.07 [1.80] | 182 | -0.35 [1.30] |
| GMFCS Level II |  |  |  |  |
| *Weight for age z-score* | 55 | -0.25 [1.47] | 56 | -0.90 [1.37] |
| *Height for age z-score* | 55 | -0.0 [1.35] | 56 | -1.05 [1.24] |
| GMFCS Level III |  |  |  |  |
| *Weight for age z-score* | 60 | -1.11 [1.65] | 57 | -1.60 [1.61] |
| *Height for age z-score* | 60 | -1.07 [1.66] | 57 | -1.67 [1.22] |
| GMFCS Level IV |  |  |  |  |
| *Weight for age z-score* | 75 | -1.50 [2.64] | 74 | -2.53 [1.94] |
| *Height for age z-score* | 75 | -1.12 [2.05] | 74 | -2.12 [1.64] |
| GMFCS Level V |  |  |  |  |
| *Weight for age z-score* | 146 | -2.55 [2.37] | 31 | -2.62 [2.06] |
| *Height for age z-score* | 146 | -1.87 [1.97] | 31 | -2.18 [2.24] |
|  |  |  |  |  |

GMFCS: Gross Motor Classification System. ^a^ There is no information of weight-for-age for children older than 10 years old from the WHO charts
